# A new perspective on the pathogenesis of chronic renal disease in captive cheetahs (*Acinonyx jubatus*)

**DOI:** 10.1371/journal.pone.0194114

**Published:** 2018-03-07

**Authors:** Emily P. Mitchell, Leon Prozesky, John Lawrence

**Affiliations:** 1 Department of Research and Scientific Services, National Zoological Gardens of South Africa, Pretoria, South Africa; 2 Department of Paraclinical Sciences, Faculty of Veterinary Science, University of Pretoria, Onderstepoort, South Africa; The University of Tokyo, JAPAN

## Abstract

The sustainability of captive cheetah populations is limited by high mortality due to chronic renal disease. This necropsy study, conducted on 243 captive cheetahs from one institution, investigated the relationships between focal palatine erosions, gastritis, enterocolitis, glomerulosclerosis, chronic renal infarcts, renal cortical and medullary fibrosis, and renal medullary amyloidosis at death. Associations between the individual renal lesions and death due to chronic renal disease and comparisons of lesion prevalence between captive bred and wild born and between normal and king coated cheetahs were also assessed. All lesions were significantly positively correlated with age at death. Renal medullary fibrosis was the only lesion associated with the likelihood of death being due to chronic renal disease, and cheetahs with this lesion were younger, on average, than cheetahs with other renal lesions. Alimentary tract lesions were not associated with amyloidosis. All lesions, except for palatine erosions, were more common in wild born than in captive bred cheetahs; the former were older at death than the latter. Having a king coat had no clear effect on disease prevalence. These results suggest that age and renal medullary fibrosis are the primary factors influencing the pathogenesis of chronic renal disease in captive cheetahs. Apart from amyloidosis, these findings are analogous to those described in chronic renal disease in domestic cats, which is postulated to result primarily from repetitive hypoxic injury of renal tubules, mediated by age and stress. Cheetahs may be particularly susceptible to acute renal tubular injury due to their propensity for stress and their extended life span in captivity, as well as their adaptation for fecundity (rather than longevity) and adrenaline-mediated high speed prey chases. The presence of chronic renal disease in subadult cheetahs suggests that prevention, identification and mitigation of stress are critical to the successful prevention of chronic renal disease in captive cheetahs.

## Introduction

Populations of free-ranging cheetahs (*Acinonyx jubatus*) are considered endangered primarily by habitat loss, habitat fragmentation and conflict with livestock and game farmers. Road traffic accidents, hunting and illegal trade also contribute to the decline in the free-ranging cheetah population[1]. Cheetahs kept in captivity spare the free-ranging population by providing animals for conservation education and insurance populations in zoos and breeding programmes[2]. However, the sustainability of captive populations is limited by high mortality due to veno-occlusive disease, lymphoplasmacytic gastritis and chronic renal disease (CRD)[1,3–6]. Renal disease is also common in captive Canadian lynx (*Lynx canadensis*), clouded leopards (*Neofelis nebulosa*), cougars (*Puma concolor*), jaguars (*Panthera onca*), ocelots (*Leopardus pardalis*), leopards (*Panthera pardus*), lions (*Panthera leo*), snow leopards (*Uncia uncia*) and tigers (*Panthera tigris*)[7,8]. Renal failure (as measured by inappetance, weakness, vomiting, progressive azotemia, dehydration, polyuria and depression) is the most frequent cause of death in cheetahs between 12 and 16 years old[3,6]. Chronic renal disease in cheetahs is a heterogeneous entity characterized by variable combinations of glomerular disease medullary amyloidosis, interstitial fibrosis, chronic renal infarcts, lymphoplasmacytic interstitial nephritis, pyelonephritis and papillary necrosis.[5,9,10]. Mixed patterns of glomerular disease are noted in most cheetahs: glomerular morphology consistent with membranous glomerulonephritis was found in 77% of captive cheetahs, 85% of which also had focal to global glomerulosclerosis, and increased mesangial matrix and membranoproliferative glomerulonephritis were also commonly seen (each 26%)[10].Chronic renal disease in cheetahs is associated with progressive irreversible loss of renal function over months or years as measured by increasing levels of azotemia[11]. Glomerulosclerosis and nephrosclerosis together were deemed responsible for 26% of deaths in North American cheetahs over 1 month old[3]. Glomerulosclerosis severity increases with age and occurs mainly in cheetahs over 7 years old[3]. Persistent hyperglycemia due to stress has been suggested as a factor in the pathogenesis of the glomerulosclerosis, due to similarities with diabetic nephropathy in humans[12] and a strong association between glomerulosclerosis and adrenal gland hyperplasia[3]. However, the typical vascular lesions of systemic hypertension and diabetes mellitus are rarely seen in cheetahs[3,5,10] and no association was found between glomerulosclerosis and three adrenal gland morphology measurements[13]. Excessive dietary protein (through the omission of non-protein carcass components), frequent feeding in captivity and a genetic predisposition have also been postulated to affect the development of glomerulosclerosis[3,9], since glomerulosclerosis is a feature of chronic progressive nephropathy in several strains of aging rats, the progression of which is sensitive to dietary protein levels[14]. Despite the presence of membranous glomerulonephritis, no evidence of immune complex deposition has been found[3,10].Renal failure was determined to be the sole or a contributing cause of death in 74% of adult North American cheetahs with medullary amyloidosis[6]. Amyloid deposits in cheetahs occur predominantly in the outer renal medulla and have been identified as the AA type[6]. Although the pathogenesis of amyloid aggregation in tissues is incompletely understood, AA amyloidosis is often associated with prolonged production of the acute-phase proteins, including serum amyloid A proteins, during chronic inflammation[15–17]. In addition to chronic inflammation, systemic amyloidosis may be associated with age[17], stress[18,19] and genetic factors[20–22]. The possibility that AA amyloidosis may be transmissible, like prion proteins, has also been explored.[23]

Deposited amyloid fibrils disrupt tissue architecture and result in progressive renal dysfunction in humans[24], domestic cats[25] and cheetahs[5,6,9]. The most common inflammatory condition in captive cheetahs is lymphoplasmacytic gastritis[5,6,9], although focal palatine erosions, lymphoplasmacytic interstitial nephritis, enterocolitis, and various other traumatic and infectious causes of inflammation are also recorded [5,9,13]. Captive cheetahs suffer from inflammation of maxillary palatine clefts that may lead to chronically inflamed focal palatine erosions and oronasal fistulae[26]. These have been ascribed to inadequate dental wear as a result of the feeding of improper diets in captivity (particularly in growing animals)[26]. However, as they occur in free-ranging animals, especially juveniles, a genetic predisposition to dental malocclusion may play a role in their development[27–29].

Gastritis is associated with *Helicobacter* spp. infection[30]. However, since free-ranging cheetahs are similarly infected but rarely show gastritis, other factors including stress are thought to account for the high prevalence of gastritis in captive cheetahs[31–33]. Strong morphological and functional evidence of stress has been demonstrated in captive but not free-ranging cheetahs[32]. Gastritis scores have been positively correlated with fecal glucocorticoids, temperament and suspected stress factors such as number of institutions in which the animal has lived, degree of public exposure, cheetah density, small enclosures and lack of exercise[33]. Affected gastric mucosa contains increased numbers of activated B cells and plasma cells, suggestive of a Th1:Th2 shift[34], which is a feature of the influence of glucocorticoid secretion in chronic stress in humans[35]. Diet in captivity also affects the incidence and prevalence of gastrointestinal tract disease in captive cheetahs[36,37]. Cheetahs are susceptible to oxalate nephrosis which is not primarily caused by ethylene glycol exposure and is not linked to concurrent glomerulosclerosis or medullary amyloidosis [38].

Given the susceptibility to CRD of captive cheetahs, the suspected role of alimentary tract inflammation in the pathogenesis of CRD and the effect of CRD on the sustainability of captive cheetah populations, we conducted a retrospective necropsy study to investigate the relationships between alimentary tract and renal lesions; the association between individual renal lesions and death due to CRD; and comparisons of lesion prevalence between captive bred and wild born and between normal coated and king coated cheetahs in 243 captive cheetahs from one institution.

## Materials and methods

Acquisition, breeding, disease and mortality records as well as pathological descriptions were examined for 243 cheetahs from a single cheetah breeding centre (1967–2014). All cases included were animals submitted for necropsy examination to investigate the cause of death. All cases included were animals submitted for necropsy examination to investigate the cause of death so an ethical statement is not relevant. The project was approved by the University of Pretoria Animal Ethics Committee (Project number VO63-14) and the National Zoological Gardens of South Africa Research Ethics and Scientific Committee (Project number P13/27). One author (E.P. Mitchell) conducted full necropsies and histological examinations of all tissues apart from the eye and spinal cord on 231 of the cheetahs between 1996 and 2014 and scrutinized the necropsy reports and, where available, histological slides from the remaining 12 cheetahs (S1 Table).

The following data were recorded: sex; date of birth (for captive bred animals) and estimated date of birth (for wild born animals); whether or not the cheetahs had normal coats or were king coat variants; and age at death. Based on published life history information[39], five age groups were identified: neonates (up to 20 days old), juveniles (21 to 83 days old), subadult cheetahs (84–810 days old), adults (811–3600 days old) and elderly cheetahs (3601+ days old). The following information was also recorded: whether or not CRD caused death; presence or absence of focal palatine erosions, enterocolitis and chronic renal infarcts at death; and scores of gastritis, glomerulosclerosis, renal cortical fibrosis, renal medullary fibrosis and medullary amyloidosis at death. Chronic renal disease was deemed to have caused death if the clinical signs and clinical pathological data submitted with the carcass were compatible with renal failure (polyuria, polydipsia, dehydration, urea >10Ummol/L, creatinine>100umol/L and urine specific gravity <1.035)[36,40]; severe renal lesions were present affecting over 60% of the renal mass; parathyroid hyperplasia and metastatic mineralisation were present; and lesions in other organs were mild or absent. Chronic renal disease was not assigned as the cause of death in cheetahs which had moderate or severe lesions in other organs. Data relating to oxalate nephrosis were excluded as previous work had demonstrated no connection between this condition and other renal parameters [38].

Histological examination was done on sections of formalin fixed paraffin embedded tissues stained with hematoxylin and eosin, von Kossa and Masson’s trichrome stains[41], and a modified PAS-Trichrome stain[42] using celestine blue instead of Weigert’s hematoxylin. Scoring of lymphoplasmacytic superficial atrophic gastritis and glomerulosclerosis was based on published accounts of these lesions in cheetahs[5,9]. The term glomerulosclerosis was retained for consistency and comparison with historical literature [3,5] in which the lesions described encompass the combination of membranous glomerulonephritis and glomerulosclerosis more recently documented in most captive cheetahs[10]. In addition, since the classification of glomerular morphology in felids is not well established[43] and kidney samples were from necropsy specimens with variable autolysis, transmission electron microscopy and detailed classification of glomerular lesions in individual animals was not attempted. Glomerulosclerosis and cortical fibrosis were evaluated in areas of the renal cortex not affected by chronic renal infarcts. Renal cortical fibrosis was evaluated as the proportion of the cortex containing collagen between tubules in fine to broad radial streaks with or without sclerotic or obsolescent glomeruli: absent (0); 1–33% (1); 34–66% (2); and 67–100% (3). Renal medullary fibrosis was evaluated as the proportion of the outer third of the medulla containing multifocal or diffuse collagen deposits between tubules: absent (0); 1–33% (1); 34–66% (2); and 67–100% (3). Although usually concordant, fibrosis of the inner medulla was not included in the parameter medullary fibrosis. Renal medullary amyloidosis was evaluated as the proportion of the outer third of the medulla containing multifocal or diffuse amyloid deposits between tubules: absent (0); 1–33% (1); 34–66% (2); and 67–100% (3). Lymphoplasmacytic interstitial nephritis was not evaluated as this multifocal usually mild lesion is considered to be secondary to glomerular or tubular injury where this is present[5,13,43]. Lesions of uremic gastropathy (gastric arteriolar necrosis, infarction, ulceration and interstitial mineralization)[43] were excluded from the parameter “gastritis”.

Statistical analyses were based on a set of ordinal variables. Hypotheses were tested using generalized linear models and included variables with binomial distributions (the presence or absence of focal palatine erosions, enterocolitis, chronic renal infarcts, and whether or not CRD caused death); variables with Poisson distributions (scores of 0–3: gastritis, medullary amyloidosis and fibrosis, glomerulosclerosis and cortical fibrosis); or a continuous (Gaussian) variable (age in days). All analyses were carried out in R 3.13 (R Core Team 2015, https://www.r-project.org/) using the library MASS for generalized linear models. In each case, models were compared using Akaike’s Information Criterion adjusted for small sample sizes (AICc)[44]. In each model set the minimum AICc value was subtracted from the value for each model to estimate the ∆AICc. Models were accepted as best-fit models if the ∆AICc score was <2[45]. For each analysis, generalized linear models were compared for best fit (∆AICc) and the statistical significance of each effect by examining generalized linear models with the relevant factors as predictors (Effect 1), generalized linear models with linked factors (additive, Effect 2) and generalized linear models with both as well as the interaction between them (Effect 3). Significance levels were set at p<0.05.

Effects and covariates included in the generalized linear models varied according to the nature of the question being examined. Four analyses were performed. Firstly, a hypothesis that alimentary tract and renal lesions result in reduced survival was tested by comparing the age at death (in days) of cheetahs with differing lesion scores. However, in all lesions, a higher score was seen in older animals, indicating a direct relationship between pathology and age at death. Therefore, lesions were treated as dependent variables on age at death for further analysis and the continuous variable age at death (in days) was used in all statistical hypotheses tested. This was necessary not only to detect an influence of age on the data, but also to account for any effects of age when testing influence/s of other variables. Secondly, generalized linear models with and without age at death as a covariate and its interaction with various lesions, were compared to examine the relationships between focal palatine erosions, gastritis, enterocolitis, medullary fibrosis and amyloidosis, glomerulosclerosis, cortical fibrosis and chronic renal infarcts. Due to autolysis obscuring details in various organs, comparisons had different sample sizes so models were run using data subsets of equal size, including only those cheetahs for which all factors present in a particular model had available data. Thirdly, to investigate the association between renal lesions and death due to CRD, the effect of each lesion (with and without age at death as a covariate) on death due to CRD was evaluated using generalized linear models for subsets of equal sample size. Lastly, two discrete variables were used as effects in generalized linear models for each lesion to indirectly assess the effect of stress and genetic factors respectively: whether cheetahs were wild or captive born; and whether cheetahs had the double recessive gene for the king coat variant or had normal coats. Age at death (Effect 1) and its interactions with the main effect (Effects 2 and 3) were included in the stepwise modeling procedure and models including both wild born and king coated cheetahs were also incorporated to test for interactive effects in these two factors.

## Results

**Fig 1:**
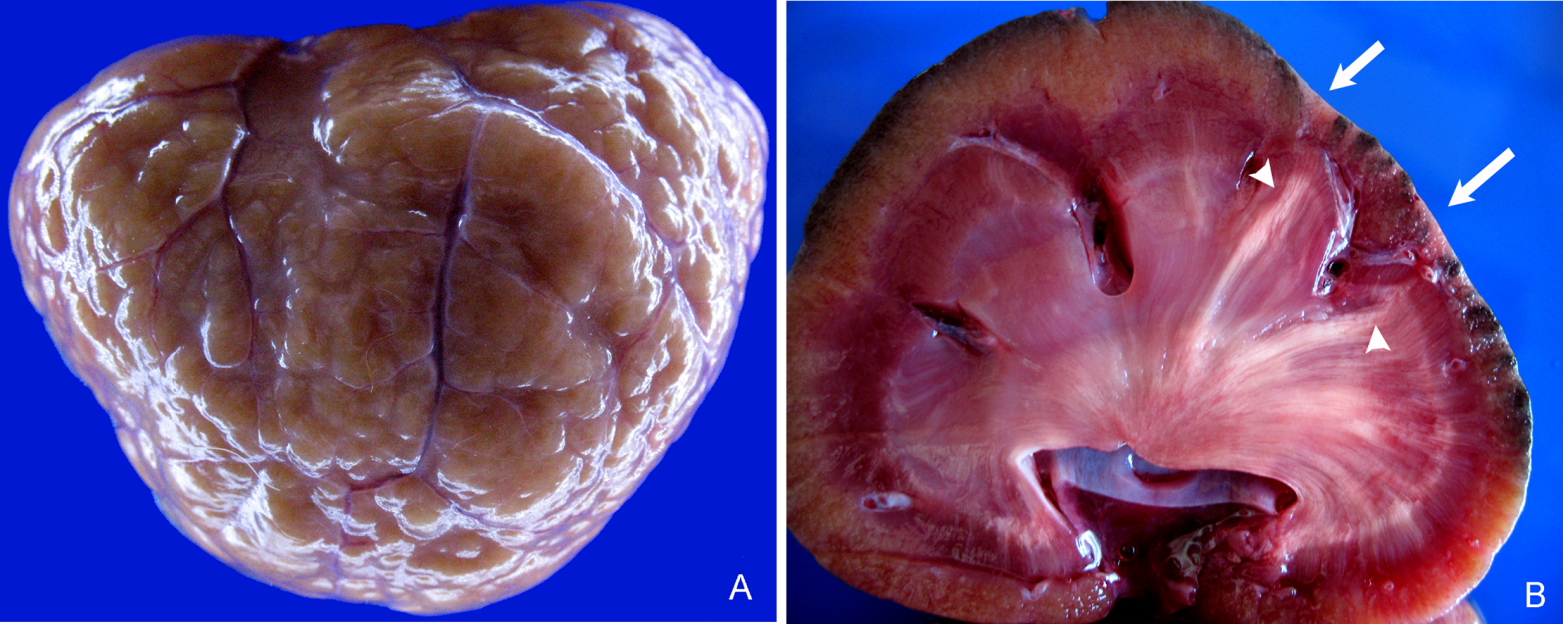
Macroscopic appearance of chronic renal disease in captive cheetahs (*Acinonyx jubatus*). A) pale misshapen kidney with a multifocally pitted and indented capsular surface due to foci of renal cortical fibrosis; B) cut section of kidney showing linear rays of renal medullary fibrosis and amyloidosis extending from the corticomedullary junction to the pelvis (arrowheads) which are variably associated with a narrowed overlying cortex due to chronic renal infarction(arrows).

**Fig 2:**
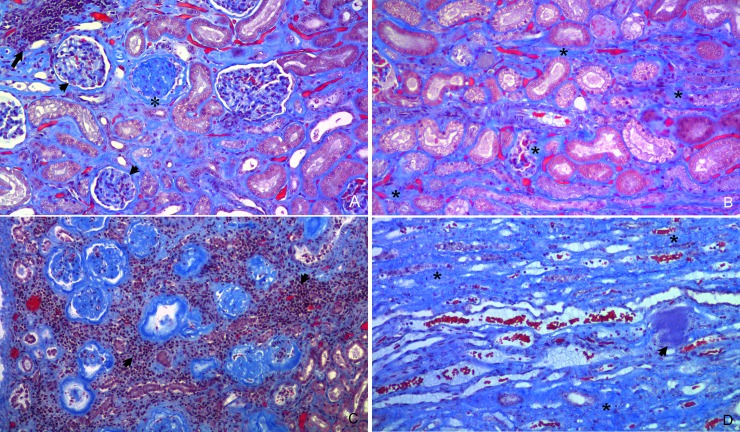
Histological appearance of chronic renal lesions in captive cheetahs (*Acinonyx jubatus*, Masson’s trichrome stain). A) glomerulosclerosis is characterized by small glomeruli with thickened glomerular basement membranes and capsules (short arrows) compared to a normal glomerulus on the right. Note one shrunken, eosinophilic, hypocellular (obsolescent) glomerulus (*),and lymphoplasmacytic inflammation (long arrow); B) diffuse cortical interstitial fibrosis consisting of blue staining collagenous material between tubular loops (*); C) chronic renal infarct characterized by clustered obsolescent glomeruli, loss of tubules and secondary lymphoplasmacytic interstitial inflammation (short arrows); D) diffuse renal medullary fibrosis consisting of blue staining collagenous material between tubules (*) which have no tubular epithelium. Note an irregular purple staining focus of amyloid in the medullary interstitium (short arrow).

Affected kidneys were small, with multifocally pitted and indented cortical surfaces (Fig 1A), with ill-defined radial pale tan streaks in the medulla that were variably associated with chronic renal infarcts in the overlying cortex (Fig 1B). Histologically, global glomerulosclerosis (Fig 2A) and cortical fibrosis (Fig 2B) occurred in multifocal fine to broad cortical rays which coalesced into chronic renal infarcts containing mainly obsolescent glomeruli and lymphoplasmacytic interstitial inflammation (Fig 2C). Renal medullary fibrosis and medullary amyloidosis were most common in the outer medulla; medullary fibrosis was more diffuse than medullary amyloidosis (Fig 2D).

The number and age of cheetahs with and without alimentary tract and renal lesions and death due to CRD are shown in Table 1. All lesions measured were significantly positively correlated with age at death (p<0.05) although variation was considerable. Enterocolitis was present in all age classes. Gastritis, focal palatine erosions and renal lesions were seen primarily in cheetahs that died as adult and elderly animals. The average age of cheetahs with gastritis, focal palatine erosions and enterocolitis was over 6.5 years; and that of cheetahs with renal lesions was over 7 years. Thirteen subadult cheetahs had medullary fibrosis and one subadult cheetah had medullary amyloidosis.

**Table 1:** Number and age of captive cheetahs (*Acinonyx jubatus*) with alimentary tract and renal lesions and that died due to chronic renal disease.

		Age group^a^						
Lesion	Score	Neonate	Juvenile	Subadult	Adult	Elderly	Total (%)^b^	Mean age^c^
**Gastritis (n = 183)**^**d**^	0	20	7	25	20	9	81 (44.3)	2.92 ±3.78
	1	0	0	7	16	9	32 (17.5)	6.81 ±4.16
	2	0	0	3	14	13	30 (16.4)	8.31 ±4.25
	3	0	2	2	19	17	40 (21.9)	8.14 ±3.97
**Focal palatine erosions (n = 168)**	0	23	10	22	46	45	147 (87.5)	5.87 ±5.05
	1	0	0	1	11	9	21 (12.5)	8.60 ±3.15
**Enterocolitis (n = 184)**	0	17	7	22	37	19	102 (55.4)	4.55 ±4.56
	1	5	3	18	27	29	82 (44.6)	6.51 ±4.77
**Medullary fibrosis (n = 221)**	0	29	12	32	23	7	103 (46.6)	2.06 ±3.20
	1	0	0	7	16	11	34 (15.4)	7.20 ±4.34
	2	0	0	4	19	25	48 (21.7)	8.95 ±3.42
	3	0	0	2	17	17	36 (16.3)	9.21 ±3.84
**Medullary amyloidosis (n = 235)**	0	29	12	46	38	7	132 (56.2)	2.42 ±3.24
	1	0	0	0	15	8	23 (9.8)	8.16 ±3.08
	2	0	0	1	13	19	33 (14.0)	9.76 ±2.99
	3	0	0	0	21	26	47 (20.0)	9.65 ±3.24
**Glomerulosclerosis (n = 214)**	0	29	12	45	61	26	173 (80.8)	3.93 ±4.20
	1	0	0	2	12	9	23 (10.8)	8.35 ±3.64
	2	0	0	0	5	7	12 (5.6)	10.32 ±3.28
	3	0	0	0	1	5	6 (2.8)	12.13 ±2.25
**Cortical fibrosis (n = 223)**	0	29	12	39	23	10	113 (50.7)	2.29 ±3.49
	1	0	0	4	34	19	57 (25.6)	7.92 ±3.67
	2	0	0	2	19	24	45 (20.2)	9.49 ±3.38
	3	0	0	0	2	6	8 (3.6)	10.24 ±3.12
**Chronic renal infarcts (n = 232)**	0	29	12	34	39	15	139 (59.9)	3.08 ±3.96
	1	0	0	2	44	49	93 (40.1)	9.20 ±3.25
**Death due to CRD (n = 236)**	0	29	12	47	57	35	180 (76.3)	2.69 ±3.70
	1	0	0	0	28	28	56 (23.7)	8.72 ±3.52

CRD: chronic renal disease

^a^Age at death: neonatal, 0–20 days; juvenile, 3–11 weeks; subadult, 3–27 months; adult, 2.25–10 years; elderly, 10+ years

^**b**^Total number (and percentage) of cheetahs that were assigned to each lesion score.

^c^Mean age at death with standard deviation

^d^The number of cheetahs examined varied as data sets were not complete for all 243 cheetahs.

**Fig 3:**
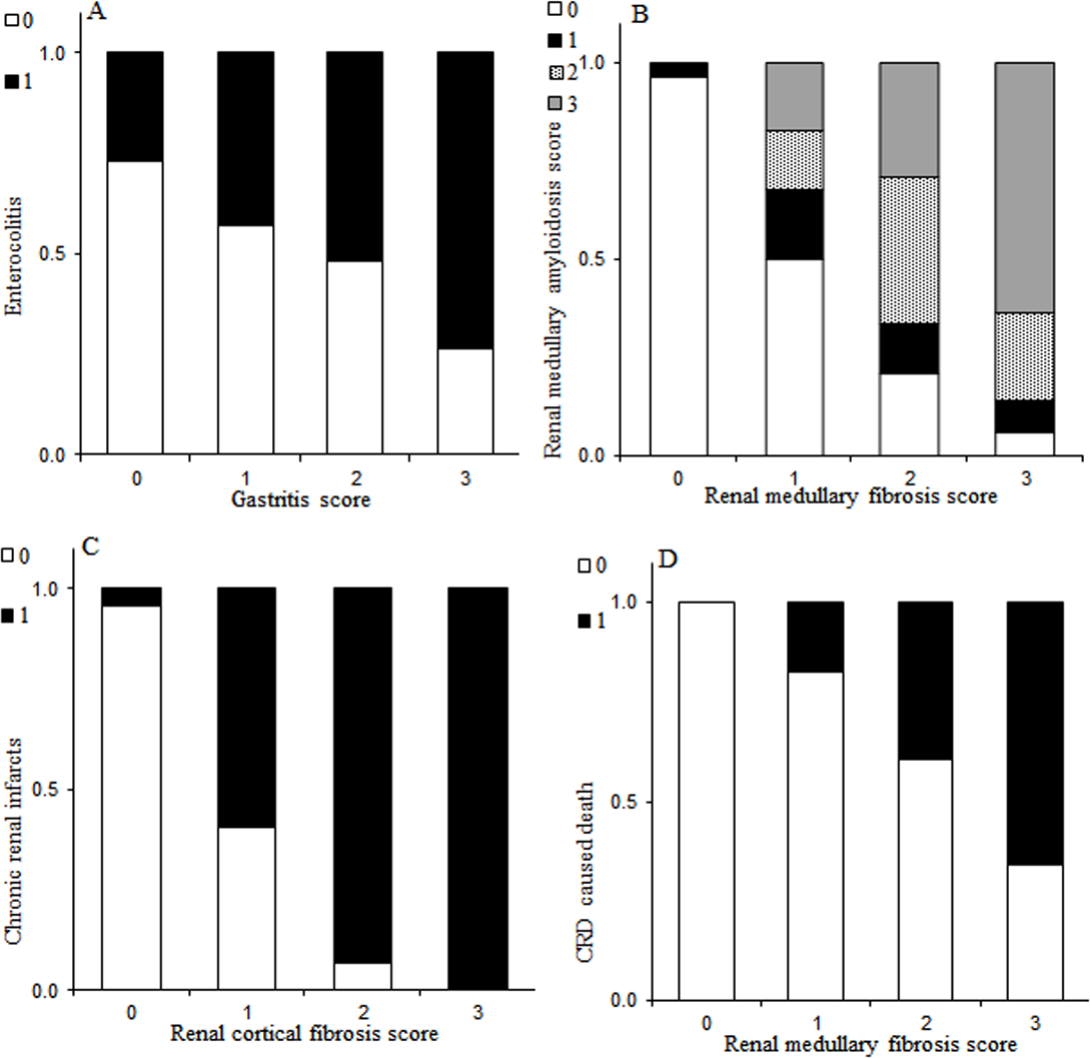
Relationships between significantly associated alimentary tract and renal lesions and death due to chronic renal disease in captive cheetahs (*Acinonyx jubatus*). The bar graphs show those associations between lesions that were statistically significant. The proportion of cheetahs with enterocolitis increased in cheetahs with higher gastritis scores. Chronic renal infarcts were rare in cheetahs without cortical fibrosis, and always present in those with severe cortical fibrosis. The proportion of cheetahs with (and severity of) medullary amyloidosis was higher in cheetahs with higher medullary fibrosis scores.None of the cheetahs without medullary fibrosis died of CRD, while the proportion of those that died of CRD increased with severity of medullary fibrosis.

The significant influence of age at death on the prevalence of all lesions documented in our study complicated the assessment of the associations between focal palatine erosions, enterocolitis, medullary amyloidosis, glomerulosclerosis and chronic renal infarcts with gastritis, cortical and medullary fibrosis. After age was taken into account, significant associations were found only between enterocolitis and gastritis, medullary amyloidosis and medullary fibrosis and between chronic renal infarcts and cortical fibrosis (Table 2). Of the 89 cheetahs with gastritis, 51 (57.3%) had enterocolitis, and this association was significant (p = 0.0011) and linear (Fig 3A). Most of the cheetahs with medullary fibrosis had medullary amyloidosis (n = 89, 75.4%); this association was significant (p≤0.0001) and linear with medullary amyloidosis score increasing with medullary fibrosis score (Fig 3B). Most of the cheetahs with cortical fibrosis had chronic renal infarcts (n = 83, 76.2%). This association was significant (p≤0.0001) and linear (Fig 3C). The prevalence of renal lesions was not significantly influenced by any alimentary tract lesion. Thirty-six cheetahs had gastritis but no medullary amyloidosis at death. Interestingly, addition of either gastritis or medullary fibrosis to the model greatly improved the model fit for glomerulosclerosis, however, neither effect was statistically significant.

**Table 2:** Best-fit (ΔAICc ≤ 2) generalized linear models testing effects of age at death (age at death, in days) and pathological lesions on the severity of different kidney lesions in captive cheetahs (*Acinonyx jubatus*).

Variable	Model	*n*	*K*	AIC_*c*_	ΔAIC_*c*_
Enterocolitis	Age at death** + gastritis**	74	3	93.26	0,00
Medullary amyloidosis	Age at death**** + medullary fibrosis**** + interaction****	85	4	192.68	0,00
Chronic renal infarcts	Cortical fibrosis****	85	2	74.09	0,00
	Age at death**** + cortical fibrosis**** + interaction*	85	4	75.17	1,09
Glomerulosclerosis	Age at death**** + gastritis	70	3	120.84	0,00
	Age at death**** + medullary fibrosis	70	3	122.79	1,95
Death due to chronic renal disease	RMF****	43	2	41.31	000

*n*: number of observations; K: number of parameters. Models including age and gastritis accounted for a significant amount of the variability in enterocolitis prevalence. Both age and medullary fibrosis (and the interaction between the age and medullary fibrosis) significantly influenced variability in medullary amyloidosis. Two models fitted for chronic renal infarcts–one where only cortical fibrosis was significant, and another where age, cortical fibrosis and the interaction between age and cortical fibrosis accounted for the variability in chronic renal infarcts. The only factor significantly affecting glomerulosclerosis variability was age; and the only factor significantly affecting whether or not cheetahs died of CRD was medullary fibrosis. Hypothesis tests for significance of effect variables

****p<0.0001

**p<0.01

*p<0.05

Of the cheetahs with CRD, medullary fibrosis was present in 53.4%, cortical fibrosis in 49.3%, medullary amyloidosis in 43.8%, chronic renal infarcts in 40.1% and glomerulosclerosis in 19.2% of animals. Chronic renal disease caused death in 56 animals. Renal medullary fibrosis was the only factor significantly associated with death due to CRD (Table 2) and no cheetahs died of CRD without having medullary fibrosis (Fig 3D). In contrast, CRD was deemed to have caused death in cheetahs without glomerulosclerosis (n = 23), chronic renal infarcts (n = 8), medullary amyloidosis (n = 4) and cortical fibrosis (n = 3).

The number and percentage of wild born, captive bred, king coated or normal coated cheetahs with each lesion and the significance of CRD are shown in Table 3. Wild born cheetahs were older at death (9.42 ± 3.65 years) than captive bred animals (4.64 ± 4.51 years) and showed greater prevalences of all lesions except for focal palatine erosions. They were more likely to die of CRD compared to captive bred cheetahs. King coated cheetahs were slightly younger at death (4.69 ± 3.97) than normal coated cheetahs (5.62 00B1 4.82). More cheetahs with the king coat had focal palatine erosions than those with normal coats but gastritis, medullary fibrosis and amyloidosis, cortical fibrosis and chronic renal infarcts were present in higher proportions of normal coated than king coated cheetahs. The proportion of king and normal coated cheetahs that died of CRD was similar. Being wild born or captive bred and having a king or normal coat were never present in the generalized linear models despite the differences noted above.

**Table 3:** Number and percentage of wild born, captive bred, normal coated or king coated cheetahs (*Acinonyx jubatus*) examined that were diagnosed with alimentary tract and renal lesions and in which chronic renal disease caused death.

Lesion present	Wild born	Captive bred	King coat	Normal coat
**Gastritis**	30/35 (85.7%)^a^	72/148 (48.7%)	8/20 (40.0%)	94/163 (57.7%)
**Focal palatine erosions**	4/39 (10.3%)	17/129 (13.2%)	3/15 (20.0%)	18/153 (11.8%)
**Enterocolitis**	20/36 (55.6%)	62/148 (41.9%)	7/17 (41.2%)	75/167 (44.9%)
**Medullary fibrosis**	35/43 (81.4%)	83/178 (46.6%)	9/21 (42.9%)	109/200 (54.5%)
**Medullary amyloidosis**	30/44 (68.2%)	73/191 (38.2%)	9/24 (37.5%)	94/211 (44.6%)
**Glomerulosclerosis**	16/39 (41.0%)	25/175 (14.3%)	4/22 (18.2%)	37/192 (19.3%)
**Cortical fibrosis**	34/43 (79.1%)	76/180 (42.2%)	9/21 (42.9%)	101/202 (50.0%)
**Chronic renal infarcts**	24/44 (54.6%)	69/188 (36.7%)	8/23 (34.8%)	85/209 (40.7%)
**Death due to CRD**	23/46 (50.0%)	33/190 (17.4%)	5/23 (21.7%)	51/213 (23.9%)

CRD: chronic renal disease.

^a^The number of cheetahs examined varied as data sets were not complete for all cases.

## Discussion

Lesion prevalence in the cheetahs described here was generally similar to that described in other cheetah populations, with some differences that may have been due to different selection criteria for animals surveyed, health management regimens and other management or environmental differences[3,5,6,9,10,26,29,36]. Evaluation of associations between lesions, the purpose of this study, was hampered by the paramount effect of age at death on lesion prevalence. Examination of the additive and interactive effects of age at death on other parameters provided a solution to this. However, the AICc statistical approach to generalized linear model selection penalises for the addition of parameters and selects models with the highest likelihood: complexity ratio, which may not be fully appropriate given the heterogeneous nature of CRD lesions in cheetahs. Therefore, factors that had a minor influence on lesion prevalence, compared to age at death, may have been excluded from the models. For example, inclusion of gastritis improved the model fit for glomerulosclerosis, suggesting that it may have a small influence on glomerulosclerosis in addition to the significant effect of by age. Confirmation of the associations between lesions, and between lesions and death due to CRD would require a prospective lifelong study of age-matched cohorts with similar environmental and management conditions, which is rarely feasible in endangered species.

Nonetheless, medullary fibrosis was the only renal lesion statistically associated with death due to CRD in this population of captive cheetahs. It was more diffuse than medullary amyloidosis, occurred in more cheetahs with CRD than any other renal lesion and no cheetah died of CRD without medullary fibrosis. In contrast, some cheetahs that died of CRD did not have medullary amyloidosis, glomerulosclerosis, cortical fibrosis or chronic renal infarcts. This effect of medullary fibrosis is consistent with studies showing that renal fibrosis is the renal lesion that most closely positively correlates with azotemia and, therefore, function of the residual renal mass in humans[46] and domestic cats[47,48]. Renal medullary fibrosis appears to precede other renal lesions in captive cheetahs because medullary fibrosis affected a higher proportion of subadult cheetahs and the average age at death of cheetahs with medullary fibrosis was approximately a year lower than those with other renal lesions.

Apart from medullary amyloidosis, which is rare in domestic cats, the macroscopic and histological findings in cheetahs in this and other[3,5,6] studies are similar to those described in CRD in older domestic cats[11,49]. Acute renal injury due to hypoxia and or toxins, exacerbated by stress and age, is thought to be a key initiating factor leading to and perpetuating CRD in cats[11,49], rats[50] and humans[51]. In an experimental model in cats[52], a single episode of renal ischemia resulted in tubulointerstitial lesions that closely resemble those described in domestic cats with CRD[49] as well as those seen in cheetahs. In our study medullary fibrosis was most severe in the outer third of the renal medulla, where relative hypoxia is greatest and metabolic activity highest[53]; repetitive subclinical hypoxic injury of renal medullary tubules may, therefore, contribute to CRD in cheetahs.

Stress is thought to play a key role in mediating tubular hypoxia in CRD in domestic cats which suffer from stress-related cystitis associated with stimulation of the sympathetic nervous system without increased adrenal steroid production[54]. In humans, dogs and rats, stress-related activation of the sympathetic nervous and renin angiotensin aldosterone (RAA) systems results in constriction of both the glomerular afferent and efferent arterioles as well as of peritubular capillaries which contributes to global glomerulosclerosis, medullary ischemia and tubular hypoxia[55,56]. Activation of the sympathetic nervous and RAA systems results in preferential perfusion of the brain, muscle and heart at the expense of other organs including the kidney and liver[57,58]. The adaptation of cheetahs to bursts of high speed locomotion may mean that they have highly active sympathetic nervous and RAA systems. Captive cheetahs have resting serum cortisol levels similar to those of domestic cats stressed by visits to veterinary hospitals, and cheetahs mount longer and higher responses to adrenocorticotrophic hormone stimulation than domestic cats[59]. Captive cheetahs also have higher stress levels than free-ranging ones[32]. Adrenocortical stress responses are well documented in captive cheetahs[60,61] and have been strongly implicated in the pathogenesis of gastritis in this species[31–33,60,62]. Stimulation of the sympathetic nervous and RAA systems as a result of chronic or repetitive stress could, therefore, play a role in the development of CRD in cheetahs.

Global glomerulosclerosis is thought to be an ischemic change in humans[63] and is the lesion seen in domestic cats[48,64] and cheetahs[3]. Inclusion of medullary fibrosis improved the model fit for glomerulosclerosis, so both lesions may be mediated by hypoxia. Cortical fibrosis and chronic renal infarcts occurred together, formed a continuum and were significantly associated in this study supporting a prior theory that they have a common pathogenesis[5]. Renal cortical lesions, regardless of their etiology, appear to be less important contributors to fatal CRD than medullary lesions in captive cheetahs since they did not contribute significantly to the risk of dying due to CRD in the generalized linear models. Similar findings have been reported in other studies[5,9]. A primary glomerular lesion is unlikely to be responsible for CRD in cheetahs since they rarely suffer from glomerular amyloid deposition, genetic kidney diseases and diabetes mellitus[5,9,10,31]. The role of immune-complex mediated glomerular disease in the development of CRD in cheetahs has not yet been clarified[3,10].

Chronic renal disease is a common age-related disease in many species including domestic cats[47,49,64], dogs[65], Island foxes[66], the great apes[67] and naked mole-rats[68]. As in humans[69], older cats have more CRD, interstitial fibrosis, sclerotic glomeruli and fewer nephrons than younger cats[48]. Age was the primary factor affecting both lesion prevalence and death due to CRD in captive cheetahs in this and other studies[3,5,6,9,13]. From an evolutionary perspective, cheetah life history emphasizes fecundity (young age of breeding, large litters) over longevity, a trade-off that may favor a shorter life span[70]. Cheetahs live significantly longer in captivity than in the wild[71] so captive cheetahs may be susceptible to age-related CRD.

Dietary factors have been postulated to play a role in the initiation and progression of CRD in cats[11,49]. Interstitial fibrosis and lymphoplasmacytic interstitial nephritis have been described in cats fed a high protein, low potassium diet[72]. Kittens fed a vitamin B_6_ deficient diet developed tubular lesions, oxalate crystals and periglomerular and peritubular fibrosis that extended radially from the corticomedullary junction[73]. Captive cheetah diets may not adequately fulfill the nutritional requirements of even healthy animals[36,37,74,75] and further research into the role of diet in the pathogenesis and the need for added requirements for cheetahs with CRD is needed.

Prior episodes of alimentary tract disease may influence the initiation and progression of CRD in humans[76] and cats[11,77]. AA amyloidosis in cheetahs is associated with chronic inflammation[6]. Although gastritis is reportedly the most common inflammatory lesion in cheetahs[5,6,9,17,31] no statistically significant association was found between alimentary tract and renal disease in this population of cheetahs. Lymphoplasmacytic nephritis but not gastritis has been shown to have a strong positive correlation with amyloidosis in cheetahs, Island foxes and Abyssinian cats [13,66,78]. Medullary fibrosis is closely associated with medullary amyloidosis in cheetahs[6,13] as well as in Abyssinian cats[78] and Shar Pei dogs[79]. Fibroblasts may act as both a nidus and a template for amyloid deposition[80] and elevated urea concentrations facilitate amyloid deposition[16]. Both these factors may explain the amyloid deposits, in cheetahs, in the outer renal medulla where medullary fibrosis occurs earlier and more extensively than the inner medulla. Medullary amyloidosis may contribute to CRD in cheetahs through exacerbation of tubular hypoxia and compromised urine concentrating ability[6]. Since not all animals or cheetahs with amyloidosis have chronic inflammatory lesions[6,19,78,81], age[19], stress[6,18,19] as well as genetic[20,21] and environmental factors such as the possibility that amyloidosis is transmissible[23] may also play a role in the pathogenesis of medullary amyloidosis in cheetahs.

The predominant effect of age at death, differing ages between groups, and unequal group sizes limited robust statistical comparison of lesion prevalence in wild born and captive bred, and king and normal coated cheetahs. Further research to determine whether these apparent differences are related to age, differing genetic diversity, or to stress levels will be hampered by the relatively small numbers of wild born and king coated cheetahs available for research.

## Conclusions

Our results suggest that CRD in our population of captive cheetahs was primarily due to medullary fibrosis. We found no evidence to support a link between alimentary tract lesions and medullary amyloidosis. Medullary fibrosis and glomerulosclerosis in cheetah may be associated with age and or stress related tubular and glomerular hypoxia. The presence of CRD in subadult cheetahs suggests that prevention, early identification and mitigation of stress may be critical to the successful prevention of CRD in this species in captivity.

## Supporting information

S1 Table Data used to evaluate the pathogenesis of chronic renal disease in captive cheetahs (*Acinonyx jubatus*). (XLSX)
